# Editorial: Computational Approaches to Study the Impact of Mutations on Disease and Drug Resistance

**DOI:** 10.3389/fmolb.2021.813552

**Published:** 2021-12-09

**Authors:** Nir Ben-Tal, Daisuke Kihara, Arun Prasad Pandurangan

**Affiliations:** ^1^ Tel Aviv University, Tel Aviv, Israel; ^2^ Purdue University, West Lafayette, IN, United States; ^3^ MRC Laboratory of Molecular Biology, Cambridge, United Kingdom

**Keywords:** mutation, genetic disorder, antimicrobial drug resistance (AMR), protein structure, computational tools and databases

Advances in next generation sequencing technologies provide wealth of data on genome variations. Understanding missense mutations is crucial to tackling global health problems related to inherited diseases and the emergence of drug resistance in cancers and infectious diseases. Advancement in research at systems and molecular level, is required to study the impact of mutations that affect both the regulation of gene expression and protein function through changes in protein stability and affinity towards other proteins, nucleic acids, biomolecules and small molecule ligands. High-quality experimental data on protein structure, mutant stability, functional annotations and phenotype-genotype associations in combination with the state of art techniques in artificial intelligence and machine will revolutionise development of highly accurate predictive computational models to study the impact of genetic mutations on human health and disease.

Predictive computational models offer an effective alternative to expensive experimental studies of genetic variations. These models can identify potential mutations linked to disease conditions and the emergence of antimicrobial drug resistance. At the molecular level proteins, via their interactions with other proteins and biomolecules, play an important role in many biological processes. The growing data on protein three-dimensional structure, along with variations observed in sequence data, will enable the development of new computational methods and tools to predict the impact of mutations on protein function, stability and interaction thereby aiding in the understanding of the basic mechanisms that govern disease conditions.

This research topic highlights the recent developments in computational approaches to analysis and predict the impact of mutation on protein stability, function and interaction. Development of accurate protein mutant stability requires the availability of properly curated high quality experimental thermodynamic dataset. To facilitate this, Turina et al. developed a semi-automatic text-mining tool to extract protein mutant thermostability data from the scientific literature. Feng et al. studied the role of phosphatase and tensin (PTEN) homolog gene mutation in low grade gliomas progression and prognosis. Using patient’s RNA sequencing data, differential gene expression and gene ontology analysis they showed that PTEN mutation promote tumorigenesis and immune cell infiltration. Tan et al. trained a predictor using saturation mutagenesis data to access the impact of point mutations on protein stability and function. Mutants are scored using a statistical potential energy function derived from protein structural data in combination with evolutionary sequence conservation and substitution scores. Using the physicochemical properties of amino acids Savojardo et al. grouped variants linked to human genetic diseases into four types and established mapping between mutations, diseases, and phenotypes through the protein family domains. Tunstall et al. designed an *in silico* framework to understand pyrazinamide resistance mutation in the clinical isolates of four main *M. tuberculosis* lineages. Using a combination of genomic features and computational mutant stability and drug affinity predictors to explain differences in modern and ancient lineages within the context of drug resistance. Using molecular dynamic simulations, Nangraj et al. investigated the mutation in *pnc*A gene of *Mycobacterium tuberculosis* that confer resistance to the first line drug pyrazinamide and explained resistance in term of the changes in protein stability and drug binding site. Prabantu et al. modelled protein structures as network where nodes and the edges correspond to residues and interaction between residues respectively. They showed that the differences between wildtype and disease mutants can be explained by their respective changes in the network both locally at the site of mutation and globally that relate to protein allosteric effects. Birolo et al. analysed both pathogenic and benign variants in haploinsufficient genes and reported that variants significantly perturbing stability (both the stabilising and destabilising) correlate with pathogenicity. Mahlich et al. performed mutational analysis using variant effect predictor on human proteins and its orthologous from 20 species. They analysed the impact of common and rare variants in terms of conservation and also suggested that cross-species variants (CSVs) might be more often neutral than non-CSVs. Bhasin and Varadarajan used large scale mutational scanning dataset to study the mutational sensitivity and substitution preferences at buried and exposed positions. They used mutational sensitivity data and predicted sequence-based accessibility values to identify buried, active-site and exposed non active-site residues. Soto-Ospina et al. aimed to understand the impact of pathogenic mutations in amyloid precursor protein Presenilin 1 that are known to cause Alzheimer’s disease. They used molecular modelling and dynamic simulations to explain the impact of mutations in terms of structural modifications of active site mutant residues found at the catalytic pore. In a focused review, Grace et al. explored the use of molecular docking and dynamics to study resistance mutation in *Mycobacterium tuberculosis* within the context of anti-tuberculosis drugs.

Understanding the impact of genetic mutation is critical to tackle disease and drug resistance. Experimental structures of biomolecules are becoming available at a rapid pace due to the recent developments in the field of cryo electron microscopy. In parallel, the technology development in computing hardware and software has enabled development of robust machine learning models to predict the structure of proteins and its interactions. Both these recent developments complement each other to provide high quality structural data of biological macromolecules and small molecules including drugs. The timing of these recent developments will enable decoding the complex mutational landscape and enable our understanding of the genotype to phenotype relationship, paving way to the achievement of precision medicine.

